# Short-term prediction of clinical and radiographic contralateral hip osteoarthritis after index total hip arthroplasty

**DOI:** 10.1007/s00402-024-05615-9

**Published:** 2024-12-12

**Authors:** Ana Ocokoljic, Lukas Krivec, Assil-Ramin Alimy, Alexander Simon, André Strahl, Frank Timo Beil, Tim Rolvien

**Affiliations:** Department of Trauma and Orthopaedic Surgery, Division of Orthopaedics, University Medical Center Hamburg-Eppendorf, Martinistraße 52, 20246 Hamburg, Germany

**Keywords:** Osteoarthritis, Arthroplasty, DXA, BMD, Hip

## Abstract

**Introduction:**

Patients with primary hip osteoarthritis undergoing unilateral total hip arthroplasty (THA) often face uncertainty about the future need for arthroplasty in the contralateral hip. We aimed to identify parameters that have predictive value with regard to the necessity for contralateral THA or the development of contralateral radiographic osteoarthritis (OA) phenotypes following index surgery.

**Materials and methods:**

In this retrospective study, we analyzed 220 patients undergoing THA. Of these, 24.1% required contralateral THA at a mean follow-up of 18.3months. Our assessments included preoperative and follow-up pelvis radiographs as well as bone mineral density (BMD) measurement by dual-energy X-ray absorptiometry prior to index THA. Comprehensive radiological measurements such as the Kellgren-Lawrence OA grade, osteophyte evaluation as well as joint shape and alignment (including alpha and CE angles) were performed.

**Results:**

We identified three indicators at the initial assessment for predicting the need for contralateral THA: higher BMI (odds ratio (OR) 1.1 [95%-CI 1.0-1.2], *p* = 0.033), higher alpha angles (> 61.5°) (OR 2.5 [95%-CI 1.0-6.3], *p* = 0.045) and the presence of multiple osteophytes (OR 2.6 [95%-CI 1.4–4.9], *p* = 0.004). Moreover, higher alpha angles were linked to more severe radiographic OA, especially osteophytosis. Higher BMD T-scores were also associated with progressive formation of multiple and large osteophytes but not joint space narrowing.

**Conclusion:**

Three factors - BMI, alpha angle, and osteophyte number - are key short-term predictors for contralateral THA after index THA. We also identified BMD as a surrogate for osteophyte formation. These findings provide novel and valuable insights for patients and surgeons regarding risks and counseling for contralateral OA and THA.

**Supplementary Information:**

The online version contains supplementary material available at 10.1007/s00402-024-05615-9.

## Introduction

Osteoarthritis (OA) is the most common degenerative joint disorder, affecting more than 500 million people worldwide [1–3]. Among various surgical options, total hip arthroplasty (THA) stands as one of the most common and successful procedures [4–6]. A key concern and question for patients undergoing THA is the potential need for arthroplasty in the “other” (i.e., contralateral) yet minimal symptomatic or asymptomatic contralateral hip. Typically, surgeons rely on classic radiographic signs of OA, but these may not definitively indicate if symptoms will develop or whether radiographic progression will occur.

Previous research indicated that patients undergoing ipsilateral THA have an increased risk of requiring contralateral THA within ten years [7, 8]. Moreover, contralateral OA was recently reported to be present in 70.4% of THA patients [9]. However, despite both hips being visualized during the initial radiographic pelvic overview, it remains unclear what specific factors drive the progression of OA and the subsequent risk for THA, highlighting a critical gap regarding predictive parameters. Some studies have suggested that joint shape abnormalities like CAM morphology could be relevant, however, the role of OA hallmarks such as joint space narrowing, and particularly the predictive value of osteophyte number and size, remains uncertain [10–13]. In addition to radiographic OA phenotypes, the role of bone mineral density (BMD) in this context remains elusive. Previous studies have suggested that there may be an association between radiographic OA and BMD as well as antiresorptive agents [14]. However, it is not known whether BMD may be suitable as a predictive parameter regarding a future contralateral THA in high-risk patients, i.e., patients who have already undergone THA [15, 16]. Specifically, osteophytes, their relationship with BMD, and their predictive value on clinical and radiographic OA progression are poorly explored.

To address these uncertainties, we investigated the role of demographic factors, hip shape, BMD, and osteophytes to determine which of these factors may differentiate whether patients already have or will develop radiographic OA phenotypes or even require THA on the contralateral hip after index THA.

## Materials and methods

### Overview of study design and patient cohort

We retrospectively screened 1,611 patients undergoing unilateral primary THA between January 2019 and December 2023 at our tertiary university medical center. Of those, we considered all patients eligible who had undergone a standardized preoperative X-ray of the pelvis and a preoperative dual-energy X-ray absorptiometry (DXA) scan. Additionally, eligibility required a postoperative X-ray of the pelvis conducted at least six months after the initial preoperative assessment. Patients were excluded if THA was performed due to secondary OA, as these have distinct etiological and progression patterns, as well as patients with pre-existing medical conditions such as rheumatoid arthritis and chronic kidney disease (glomerular filtration rate < 30 ml/min/1.73 m²) due to their potential confounding effect on BMD associations. Additionally, patients on medications affecting bone metabolism, including anti-osteoporosis drugs or high-dose corticosteroids (≥ 7.5 mg/day of prednisolone or equivalent), were also excluded.

Based on these criteria, a total of 220 patients were included for analysis in this retrospective study. At a mean follow-up of 18.3 months, 24.1% (53 patients) subsequently became severely symptomatic on the contralateral side, i.e., the indication for THA surgery was established. In contrast, 75.9% (167 patients) showed no symptomatic progression of OA on the opposite side. Our study cohort’s overall characteristics, encompassing demographics, radiographic parameters, and DXA data, are detailed in Table 1. Our study group consisted of 132 women (60%) and 88 men (40%). The majority were older than 65 years (69.0 ± 10.7) and overweight (BMI: 28.7 ± 5.4). The study was approved by the local ethics committee, ensuring that the retrospective analysis of existing patient data adhered to ethical guidelines and the Declaration of Helsinki for patient privacy and data confidentiality.

**Table 1:** Demographics, radiographic characteristics and DXA data in the overall study cohort (*n* = 220) and stratified by the need for contralateral THA

	Total	Asymptomatic (no contralateral THA)	Symptomatic (subsequent contralateral THA)	*p*-value
	Mean (SD) or n (%)	Mean (SD) or n (%)	Mean (SD) or n (%)	
n	220	167	53	
Follow-up (months)	18.3 (12.1)	17.6 (11.8)	20.5 (12.7)	0.062
Sex (w/m)	132/88 (60.0/40.0)	108/59 (64.7/35.3)	24/29 (35.3/54.7)	**0.012**
Age (years)	69.0 (10.7)	69.5 (10.1)	67.4 (12.3)	0.223
BMI (kg/m^2^)	28.7 (5.4)	28.5 (5.6)	29.3 (5.0)	0.296
Kellgren-Lawrence grade (ipsilateral, operated side)	2.8 (0.6)	2.8 (0.6)	3.0 (0.5)	**0.002**
Kellgren-Lawrence grade (contralateral side) t_0_	2.3 (0.5)	2.2 (0.5)	2.4 (0.5)	0.131
Kellgren-Lawrence grade (contralateral side) t_1_	3.0 (0.5)	2.9 (0.5)	3.0 (0.5)	0.457
Alpha angle (°)	53.0 (8.8)	51.7 (8.4)	57.2 (9.0)	**< 0.001**
CE angle (°)	34.6 (5.8)	34.4 (5.4)	35.3 (7.0)	0.313
Osteophyte number	1.7 (0.8)	1.6 (0.7)	2.2 (0.8)	**< 0.001**
Osteophyte size (mm^2^)	46.9 (39.53)	45.0 (39.9)	71.8 (67.3)	**0.001**
**DXA**				
T-score_hip_	-0.3 (1.3)	-0.3 (1.3)	-0.3 (1.5)	0.994
T-score_min_	-0.6 (1.4)	-0.6 (1.4)	-0.6 (1.4)	0.980
Osteoporosis (T-score ≤ -2.5)	19/220 (8.6)	15/167 (9.0%)	4/53 (7.5%)	0.900
Osteopenia (-1 > T-score > -2.5)	70/220 (31.8)	52/167 (31.1%)	18/53 (34.0%)	
Normal BMD (T-score ≥ -1)	131/220 (59.5)	100/167 (59.9%)	31/53 (58.5%)	

Abbreviations: SD: standard deviation, THA: total hip arthroplasty, w: women, m: men, BMI: body mass index, CE: center-edge, DXA: dual-energy X-ray absorptiometry, BMD: bone mineral density

### Radiographic measurements

Standardized weight-bearing anteroposterior (AP) calibrated radiographs of the pelvis were obtained at baseline preoperatively and at regularly scheduled follow-up examinations in our clinic. To investigate factors associated with progression, an analysis was conducted on the contralateral hip by examining the preoperative baseline (t_0_) and the latest available follow-up (t_1_) X-ray images. During the initial evaluation for primary THA and during the follow-up examination, a range of radiological measurements were performed on the contralateral hip. The Kellgren-Lawrence (K-L) OA grade (1 to 4) and joint space width (JSW) were determined using a standardized approach [17]. The alpha and center-edge (CE) angle were determined as previously described [18–20]. Analysis of osteophytes involved quantifying both their number and area. Each distinct osteophyte was quantified using a standardized approach. Osteophytes were individually traced, and the total area (mm^2^) was determined. Measurements were performed by using ImageJ (National Institutes of Health, Bethesda, MD, USA).

### Bone mineral density

To determine areal bone mineral density (aBMD) (g/cm^2^) and corresponding Z- and T-scores, DXA was performed prior to index THA on both proximal femurs and the lumbar spine (L1-L4) using a Lunar Prodigy enCore 2007 system (GE Healthcare, Madison, WI, USA). In addition to T-scores of the contralateral hip related to index THA (T-score_hip_), we also used the lowest T-score of any measured site (T-score_min_) for further analysis.

### Data analyses

Continuous variables are presented as mean ± standard deviation (SD), while categorical variables are reported as numbers and percentages. The normality of data distribution was assessed using the Shapiro-Wilk test. Group differences were assessed for significance using an unpaired two-tailed *t*-test for normally distributed data and the Mann-Whitney *U* test for non-normally distributed data. One-way analysis of variance (ANOVA) with Tukey’s post hoc analysis for multiple comparisons was used to examine differences between more than two groups under the assumption of normal distribution of data. In the presence of non-normally distributed data, the Kruskal-Wallis test was used, followed by Dunn’s post hoc test for multiple comparisons. Differences between two categorical variables were assessed using the Chi-squared test. To identify potential predictors of progressing OA of the hip, all patients were subjected to a regression analysis. As the requirement for future contralateral THA might arise not only from radiographic features but also from other factors, including demographics, it is essential to identify baseline data that might reveal associations with the need for subsequent THA. To further understand the interplay of various factors in patients undergoing unilateral THA and subsequent development of contralateral OA, a multiple logistic regression analysis was undertaken to assess if the variables age, body mass index (BMI), sex, Kellgren-Lawrence score, alpha angle, the number and size of osteophytes, and T-scores (hip) of the contralateral side are predictive of the necessity for future contralateral THA in the patient population being studied. Because of varying alpha angle thresholds in existing studies [19, 21, 22], ranging between 50° and 60°, we defined a specific threshold based on our population for the alpha angle at 61.5°. This value was determined using Youden’s *J* statistic [23]. Youden’s *J* is a measure that uses receiver operating characteristic (ROC) curves to determine the appropriate threshold to distinguish between patients requiring a subsequent contralateral THA and those who do not. The regressions use the “Enter” method to examine the significant impact of all variables simultaneously. The analysis was conducted as a complete case analysis with no missing variables. All analyses were performed using SPSS version 29.0 (IBM, Armonk, NY, USA) and GraphPad Prism version 9.5 (GraphPad Software, La Jolla, CA). Statistical significance was set to a two-tailed *p*-value of 0.05. Exact *p*-values for statistically significant comparisons are reported unless *p* < 0.001. All data are presented as absolute values, mean alongside standard deviation (SD). Sample size calculation was performed using G*Power [24]. For sample size calculation, we assumed a medium effect size *d* of 0.8, using an allocation rate of 0.3 based on findings from a prior study regarding the alpha angle [25]. Considering an α of 0.05 and aiming for a statistical power of 0.8, we determined that a total sample size of 88 patients was required (two-tailed *t*-test).

## Results

### Higher alpha angles are associated with more severe radiographic osteoarthritis in the contralateral hip

**Fig. 1:**
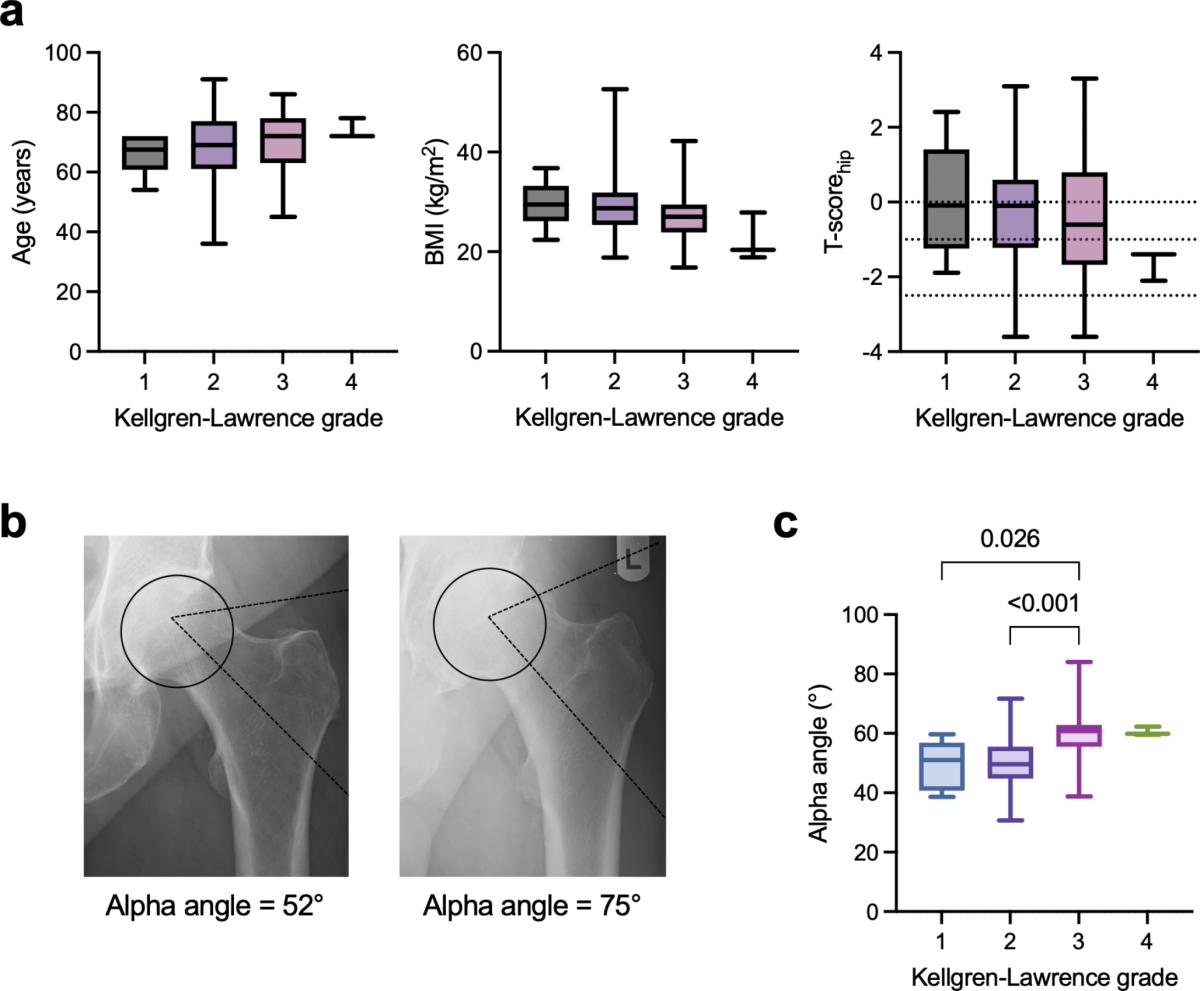
Higher alpha angles are associated with increased radiographic osteoarthritis severity of the contralateral hip. **a** Demographics and T-scores stratified by Kellgren-Lawrence grades. **b** Representative radiographs highlighting an average (52°, left image) and increased (75°, right image) alpha angle. **c** Alpha angle in relation to the Kellgren-Lawrence grades. One-way analysis of variance (ANOVA) with Tukey’s multiple comparison test was used for normal distributed data and Kruskal–Wallis test with Dunn’s multiple comparison test was used for nonparametric data. Exact *p*-values are reported for statistically significant comparisons unless *p* < 0.001. Abbreviations: KL: Kellgren-Lawrence

The evaluation of radiographic OA severity in the contralateral hip, as measured by K-L grades, revealed that most patients fell within grades 2 to 3, indicating a moderate level of joint degeneration (Suppl. Table 1). A detailed analysis of factors potentially influencing the radiological severity of contralateral OA at initial presentation revealed no differences across various parameters. Specifically, age, BMI, and T-scores (both the lowest T-score and the T-score of the hip) showed no association with K-L grade (Fig. 1a, Suppl. Figure 1). Thereafter, we shifted our focus to anatomical indicators of biomechanical joint alignment (i.e., hip shape). Here, the analysis of the CE angle revealed no link with radiographic OA severity (Suppl. Figure 2). However, we observed that higher alpha angles were associated with more severe OA (Fig. 1b). Specifically, regarding K-L grades, alpha angles were higher in patients with K-L grade 3 compared to grade 2 (*p* < 0.001) (Fig. 1c). A similar trend was observed when comparing K-L grade 3 to grade 1 (*p* = 0.026) (Fig. 1c).

### Osteophyte number and size are linked to higher alpha angles

**Fig. 2:**
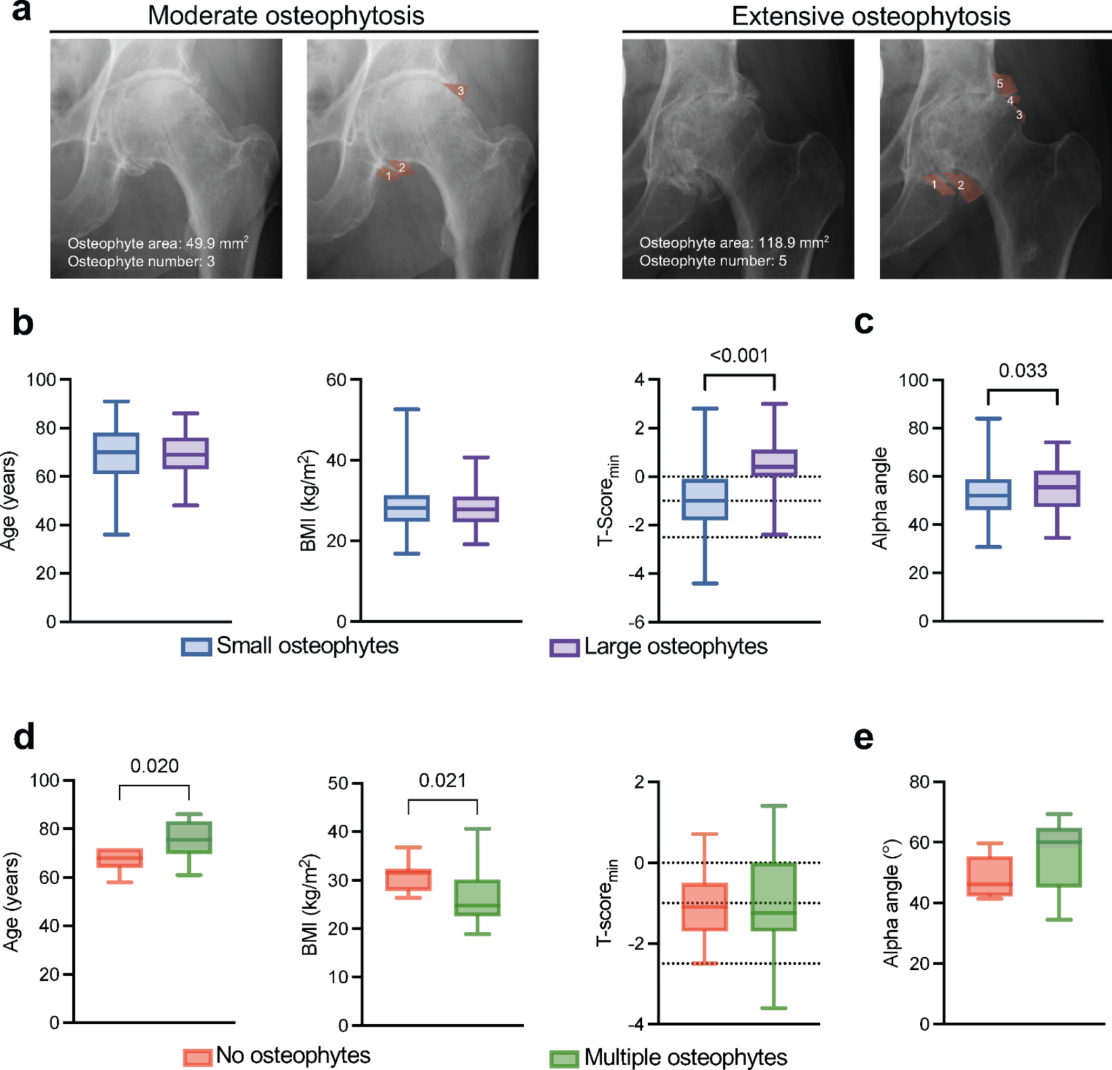
Osteophytosis is associated with higher alpha angles and higher BMD T-scores. **a** Representative anteroposterior radiographs of hip joints contralateral to index THA, with osteophytes marked in red. Left panel displays three osteophytes, while the right panel shows five osteophytes, highlighting a higher count and area. **b** comparison of age, body mass index (BMI), T-score, and **c** alpha angle between patients with small and large osteophytes. **d** Comparison of age, BMI, T-score, and **e** Alpha angle between patients with no and multiple osteophytes. Student’s *t*-test was used for normal distributed data and Mann-Whitney *U* test was used for nonparametric data. Exact *p*-values are reported for statistically significant comparisons unless *p* < 0.001

Considering that osteophytes are a key feature of OA, our analysis subsequently focused on evaluating the impact of both size and number of osteophytes on contralateral OA progression (Fig. 2a). The analysis revealed that specifically the presence of large osteophytes– defined as those exceeding the 75th percentile in size– was not associated with increased age, BMI, when compared to small osteophytes (those below the 75th percentile) (Fig. 2b). However, both higher T-scores (*p* < 0.001) and higher alpha angles (*p* = 0.033) were associated with large osteophytes (Fig. 2c). Notably, the presence of multiple osteophytes, defined as having at least three, was associated with advanced age and lower BMI. However, neither T-scores nor alpha angles showed a significant association with the occurrence of multiple osteophytes (Fig. 2d, e).

### Independent predictors for contralateral THA

The study cohort showed no significant differences regarding age and BMI between patients who underwent subsequent contralateral THA and those who did not (Table 1). However, patients who were indicated for contralateral THA had radiographically more severe OA on the ipsilateral side and were more likely to be men. Regarding radiographic OA severity on the contralateral side, there were no differences in terms of K-L grades between both groups at the initial presentation (*p* = 0.131) and at the latest available follow-up (*p* = 0.457). Moreover, patients undergoing subsequent THA exhibited higher alpha angles on the contralateral side (*p* < 0.001), while CE angles did not differ between the two groups (*p* = 0.313). Both higher numbers and greater sizes of osteophytes of the contralateral hip were found in patients requiring subsequent THA (*p* < 0.001 and *p* = 0.001). When assessing T-scores and the fraction of patients categorized as having osteoporosis, osteopenia, and normal BMD, there were no differences between both groups. Employing a multiple logistic regression model, three independent predictors for the development of contralateral OA could be revealed in this patient group (Table 2). Namely, higher BMI (odds ratio (OR) 1.1 [95%-CI 1.0 to 1.2], *p* = 0.033), higher osteophyte numbers (OR 2.6 [95%-CI 1.4 to 4.9], *p* = 0.004), and higher alpha angles (> 61.5°) (OR 2.5 [95%-CI 1.0 to 6.3], *p* = 0.045) of the contralateral hip increased the odds of a future THA.

**Table 2:** Model summary of the multiple logistic regression for independent predictors of contralateral THA

							95% CI	
Predictor	β	SE β	Wald’s χ^2^	*df*	*p-*value	OR	Lower	Upper
Constant	-3.80	1.81	4.42	1	0.035	0.02	NA	NA
Age	-0.03	0.02	2.30	1	0.130	0.97	0.94	1.01
**BMI**	**0.08**	**0.04**	**4.52**	**1**	**0.033**	**1.08**	**1.01**	**1.17**
Sex (f/m)	0.69	0.38	3.38	1	0.066	2.00	0.96	4.16
**Osteophyte number**	**0.95**	**0.33**	**8.39**	**1**	**0.004**	**2.58**	**1.36**	**4.89**
Osteophyte area	0.01	0.01	0.01	1	0.923	1.00	0.99	1.01
**Alpha angle**	**0.93**	**0.46**	**4.02**	**1**	**0.045**	**2.53**	**1.02**	**6.28**
T-score_hip_	-0.06	0.14	0.17	1	0.676	0.94	0.71	1.25
Test			χ^2^	*df*	*p-*value			
Overall model evaluation Omnibus-test			35.88	7	< 0.001			

Regression with “Enter”-method. Cox and Snell R² = 0.16; Nagelkerkes R² = 0.24. CI = confidence interval; df = degrees of freedom; OR = odds ratio, SE = standard error

### Sex-specific analysis

We observed consistent patterns across men and women. No differences were noted in age, BMI, and alpha angle across men and women (Suppl. Figure 3a-c). Women had lower T-scores in the hip (*p* = 0.004) and overall (*p* = 0.017) compared to men (Suppl. Figure 4). When analyzing the contralateral hip at baseline, women classified as large osteophyte formers did not differ in age, BMI, or alpha angle from small osteophyte formers (Suppl. Figure 5a). However, larger osteophytes in women were associated with higher T-scores (*p* < 0.001) (Suppl. Figure 5a). Although similar age, BMI and T-scores were observed in men, large osteophyte formers had higher alpha angles (*p* < 0.001) (Suppl. Figure 5b). Women with multiple osteophytes were older (*p* = 0.020) and had a lower BMI (*p* = 0.021) (Suppl. Figure 5c). While these associations were not found in men, men with multiple osteophytes also exhibited higher T-scores (*p* = 0.043) and higher alpha angles (*p* = 0.016) (Suppl. Figure 5d). JSW analyses revealed that lower JSW was associated with higher age in women (*p* = 0.030) but not in men (Suppl. Figure 5e-f). Moreover, a comparison of T-scores between those with a wide and narrow JSW indicated no differences among women and men (Suppl. Figure 5e-f). Alpha angles were higher across both women (*p* = 0.013) and men (*p* < 0.001) with narrow JSW (Suppl. Figure 5e-f).

### High BMD is associated with osteophyte formation but not loss of joint space

**Fig. 3:**
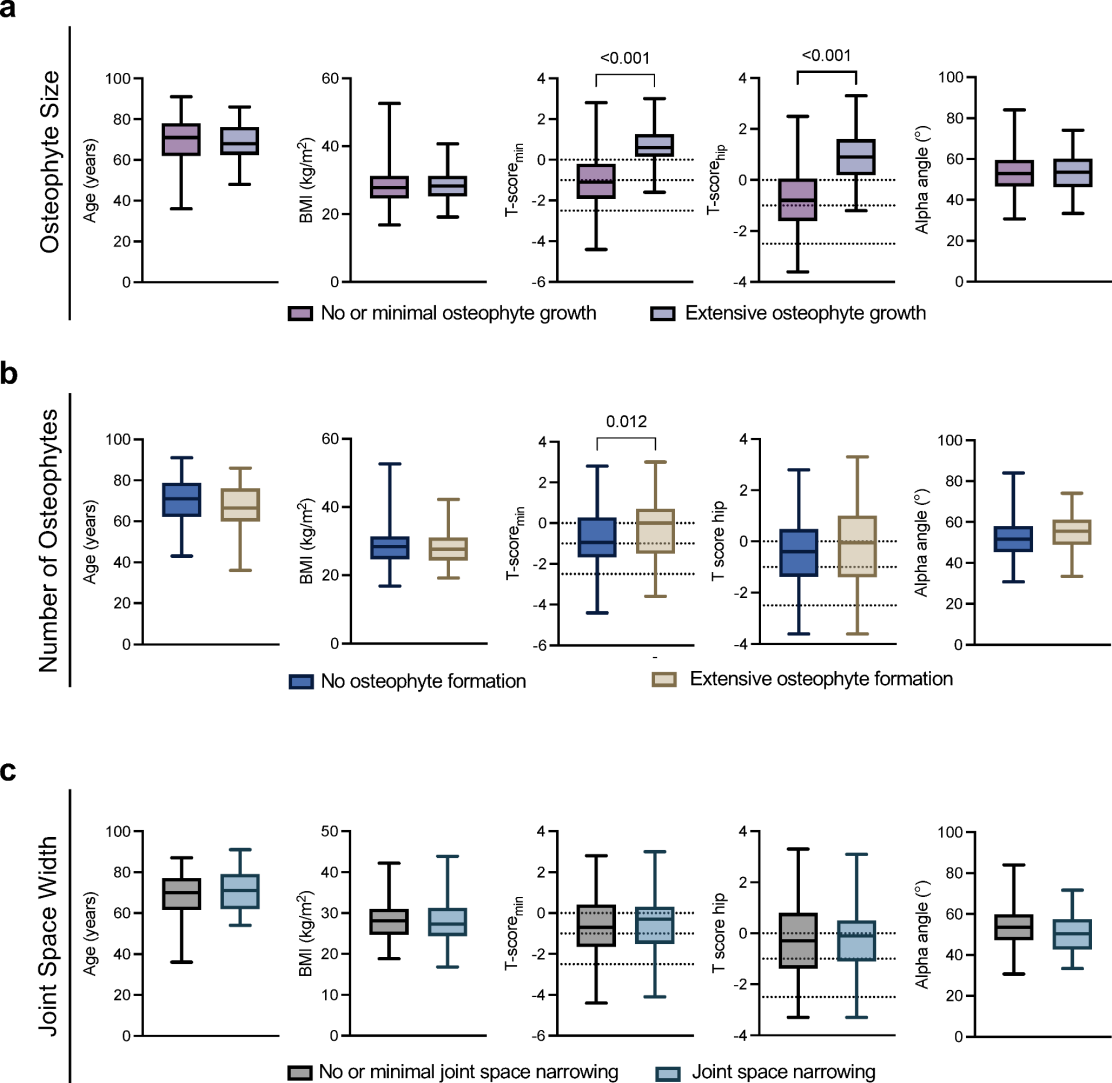
Radiographic progression of osteophytosis is linked to increased BMD. **a** Comparison of age, body mass index (BMI), T-scores, and alpha angle based on the progression of osteophyte size, **b** osteophyte number, and **c** joint space width narrowing. Student’s *t*-test was used for normal distributed data and Mann-Whitney *U* test was used for nonparametric data. Exact *p*-values are reported for statistically significant comparisons unless *p* < 0.001. Abbreviations: JSW: joint space width

In our analysis of OA progression over an average follow-up of 18.3 ± 12.1 months, we evaluated factors driving the progression of key OA features from the initial presentation for index THA to the most recent follow-up visit. Patients categorized as large osteophyte formers, who formed particularly large osteophytes, i.e., size over the 75th percentile, did not show differences in age or BMI compared to those with small osteophyte development i.e. under the 75th percentile (Fig. 3a). However, large osteophyte formers demonstrated higher T-scores than their counterparts with small osteophyte growth (Fig. 3a). Regarding newly built osteophytes, those falling into the category of multiple osteophyte formers, with at least three newly developed osteophytes, did not differ in terms of age, BMI, or alpha angle (Fig. 3b). However, they also exhibited higher T-scores compared to patients with no osteophyte formation (*p* = 0.012) (Fig. 3b). Regarding JSW, patients with extensive joint space narrowing, did not differ in age, BMI, T-score and alpha angles from those with no joint space narrowing (Fig. 3c).

## Discussion

In this study, we addressed the critical question of how to estimate the need for contralateral THA in high-risk patients, i.e., patients who underwent unilateral index THA. Patients undergoing unilateral THA often ask about the prognosis of the contralateral hip, seeking to understand the potential future need for contralateral THA. This question, while commonly asked, presents a challenge for orthopedic surgeons due to the complexity of estimating OA progression and, thus, the need for contralateral THA. Our study involved analyzing early radiographic indicators in pelvic overview radiographs at the initial presentation for index THA to provide evidence-based answers to a common patient concern regarding the “other”, yet minimal symptomatic or asymptomatic, contralateral hip to improve patient counseling.

Comparisons of parameters measured at the initial presentation showed that patients who required contralateral THA had greater alpha angles of the contralateral hip and increased K-L grades of the ipsilateral hip. Our multiple logistic regression model builds upon this understanding and allowed us to identify three critical and independent predictors (BMI, alpha angle, and osteophyte number) for future contralateral THA. Another intriguing aspect was that higher BMD T-scores were associated with the progression of radiographic OA phenotypes. Specifically, this progression was mainly characterized by the formation of multiple osteophytes (i.e., osteophytosis).

While previous studies have made strides in addressing the question of contralateral THA prediction, definitive clarity remains elusive, particularly with regard to novel radiological parameters and BMD [7, 10, 12, 13]. Our analysis is consistent with previous studies regarding the impact of the BMI and alpha angle [12, 26]. While the alpha angle proved to be a significant predictor for subsequent THA, the CE angle did not demonstrate such predictive value, consistent with findings from previous research [12]. In our study, we observed that factors like age did not differentiate between patients who later required contralateral THA and those who did not. This finding challenges some conventional assumptions, underscoring the complexity of OA progression [27]. However, our analysis reaffirmed the role of obesity as a differentiating factor regarding future contralateral THA [26, 28–30].

A notable finding of our study is the relevance of the alpha angle in contralateral OA estimation. Patients with higher alpha angles on the contralateral side demonstrated elevated K-L grades, indicative of more pronounced OA. This also fits our results that larger sizes osteophytes were linked to higher alpha angles, indicating a potential relationship between an abnormal femoral head-neck junction and the occurrence of multiple osteophytes. Even within our relatively short follow-up period, higher alpha angles emerged as early indicators for impending THA. This underlines the established connection between increased alpha angles and severe radiographic OA, suggesting that high alpha angles may accelerate progression to end-stage OA, necessitating THA [25, 31]. This was also underscored by our regression model, which confirmed its viability as a suitable predictor of future contralateral THA.

Another novel aspect of our study is the role of osteophytes as predictors for requiring contralateral THA. Specifically, our findings suggest that individuals with multiple osteophytes on the contralateral side at the time of initial presentation for index THA are at an increased risk for requiring contralateral THA in the future. Interestingly, our data also revealed that patients with multiple osteophytes at initial presentation and progressive osteophytosis during the follow-up period had higher BMD T-scores. This finding underlines the potential link between high BMD and osteophytes, which has been previously discussed in the context of knee OA [16, 32]. High BMD may suggest a tendency towards more active bone formation, which could ultimately lead to an increased OA risk. Conversely, previous translational work has shown that certain genes, among others the mechanically activated ion channel Piezo1, whose loss of function is associated with reduced bone mass, are also linked to reduced osteophyte formation [33]. Thus, high DXA T-scores may be indicative of multiple, large osteophytes, proposing BMD as a surrogate marker for osteophyte formation. This hypothesis aligns with previous research suggesting osteophytes as an early indicator of OA, often preceding joint space narrowing and being asymptomatically [34]. Ultimately, this finding underscores the value of assessing both osteophytes and BMD with regard to future contralateral THA.

Our study has a few limitations, including a relatively small sample size and a retrospective study design. However, this is the first study to incorporate analyses of osteophytes and BMD in relation to contralateral OA and future THA needs, particularly in high-risk patients. Our study differs from previous research due to its relatively short follow-up period [7]. While this represents a limitation, it also uniquely highlights parameters associated with rapid OA progression and the need for THA. Therefore, future prospective studies, should be conducted and include regularly scheduled follow-up examinations with radiographic analyses of osteophytes to more accurately assess their value in predicting OA progression in a prospective setting over longer time periods. Furthermore, our exclusion criteria introduce limitations by excluding patients with secondary OA and those with severe comorbidities such as CKD. Thus, our findings primarily apply to a healthier cohort with primary OA. While this approach ensures a relatively homogeneous cohort and enhances applicability to the majority of patients, future studies should conduct subgroup analyses to explore whether our findings can be confirmed in other patient groups.

Another limitation of our study is that the study sample was limited to patients who had undergone preoperative DXA scan. Since DXA is generally only performed in patients who have risk factors for osteoporosis, our study may not be applicable to the entire population of patients with hip OA. However, most of the patients in this study were over 70 years of age, and in these patients, the indication for a DXA examination is usually given regardless of additional risk profiles.

In conclusion, our study identified three key risk factors - BMI, alpha angle, and osteophyte number - as having independent predictive value for contralateral symptomatic OA in patients presenting for index THA. We also identified BMD as a potential surrogate for osteophyte formation. Consequently, surgeons may consider analyzing these parameters during the initial presentation to improve patient-centered care and counseling regarding future contralateral THA.

## Electronic supplementary material

Below is the link to the electronic supplementary material.

Supplementary Material 1

## Data Availability

The data that support the findings of this study are available from the corresponding author upon reasonable request.
