# Supplementary material for: Short-term prediction of clinical and radiographic contralateral hip osteoarthritis after index total hip arthroplasty
Source: Arch Orthop Trauma Surg. 2024 Dec 12;145(1):7. doi: 10.1007/s00402-024-05615-9 (PMC11638392; doi:10.1007/s00402-024-05615-9)
Supplement: Supplementary file 1 — Supplementary Material 1 [file 402_2024_5615_MOESM1_ESM.docx]

Supplementary Information for

**Short-term prediction of clinical and radiographic contralateral hip osteoarthritis after index total hip arthroplasty**

**Supplementary Tables**

**Supplementary Table 1:** Distribution of Kellgren-Lawrence grades of the contralateral hip.

| Kellgren-Lawrence (K-L) Grade | Total (n, %) | Asymptomatic  (no contralateral THA) (n=167), (n, %) | Symptomatic (subsequent contralateral THA) (n=53), (n, %) | | *p*-value |
| --- | --- | --- | --- | --- | --- |
| K-L Grade 1 | 6 (2.7) | 6 (3.6) | | 0 (0) | **0.176** |
| K-L Grade 2 | 151 (68.6) | 117 (70.1) | | 34 (64.2) |  |
| K-L Grade 3 | 60 (27.3) | 41 (24.6) | | 19 (35.8) |  |
| K-L Grade 4 | 3 (1.4) | 3 (1.8) | | 0 (0) |  |

Abbreviations: THA: total hip arthroplasty.

**Supplementary Figures**

**
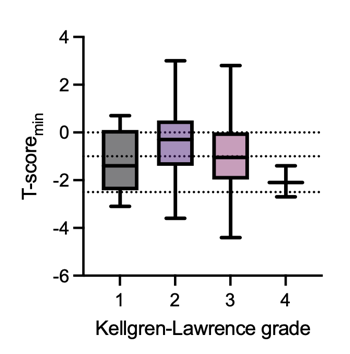
**

**Suppl. Fig. 1: Limited association of T-score_min_ and radiographic OA severity.**

T-score_min_ corresponding to Kellgren-Lawrence grades. One-way analysis of variance (ANOVA) with Tukey's multiple comparison test was used for normal distributed data and Kruskal–Wallis test with Dunn’s multiple comparison test was used for nonparametric data. Exact *p*-values are reported for statistically significant comparisons unless *p*<0.001.


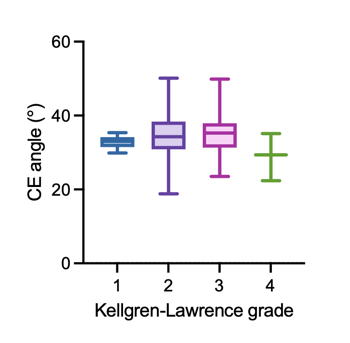


**Suppl. Fig. 2: Limited association between Center-edge angle and radiographic OA severity.**

Distribution of center-edge angles across Kellgren-Lawrence grades. One-way analysis of variance (ANOVA) with Tukey's multiple comparison test was used for normal distributed data and Kruskal–Wallis test with Dunn’s multiple comparison test was used for nonparametric data. Exact *p*-values are reported for statistically significant comparisons unless *p*<0.001. Abbreviation: CE: center-edge


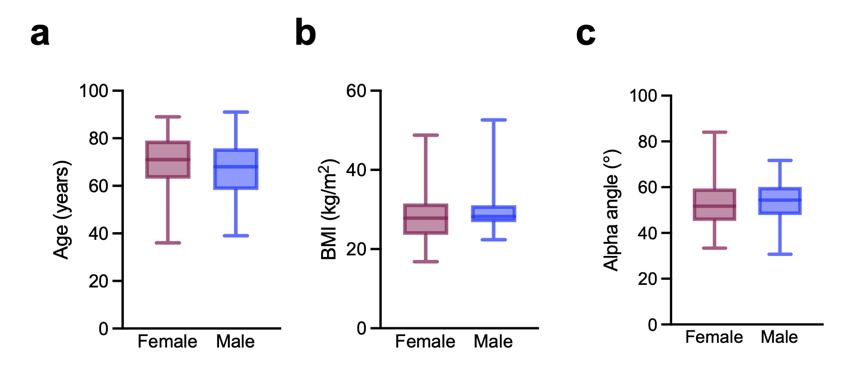


**Suppl. Fig. 3: No relevant sex-specific differences across age, BMI and alpha angle.**

**a** Comparison of mean age between women and men indicates no difference. **b** Evaluation of body mass index (BMI) reveals no difference between women and men. **c** Comparison of alpha angle shows no difference in women and men. Student’s *t*-test was used for normal distributed data and Mann-Whitney *U* test was used for nonparametric data. Exact *p*-values are reported for statistically significant comparisons unless *p*<0.001.


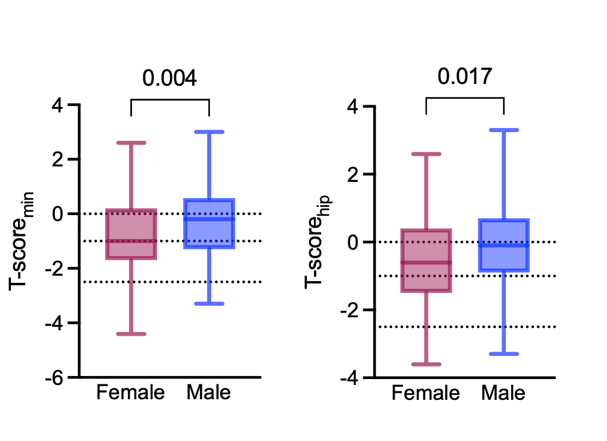


**Suppl. Fig. 4: Sex-specific differences are limited to a lower bone mineral density (BMD) in women.**

Dual-energy X-ray absorptiometry measurements reveals lower BMD T-score_min_ (left panel) and T-score_hip_ (right panel) in women compared to men. Student’s *t*-test was used for normal distributed data and Mann-Whitney *U* test was used for nonparametric data. Exact *p*-values are reported for statistically significant comparisons unless *p*<0.001.

**
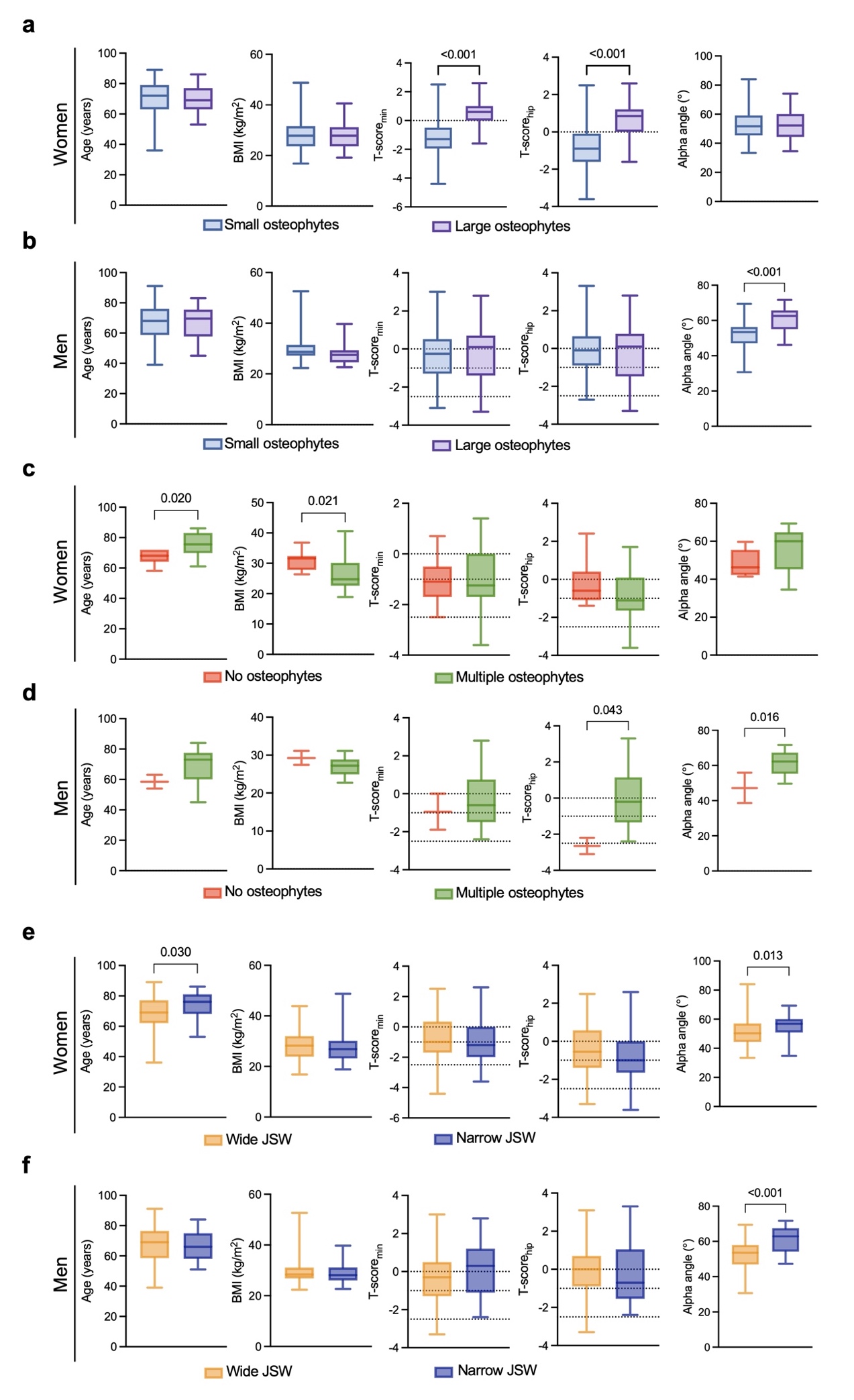
**

**Suppl. Fig. 5: Sex-specific evaluation of various baseline parameters of the contralateral hip in relation to osteophyte size, number, and joint space width.**

**a** Analysis of age, body mass index (BMI), T-scores and alpha angle reveals no difference between women with extensive and limited osteophyte development. **b** Evaluation of demographic parameters including age and BMI, as well as dual-energy X-ray absorptiometry evaluation of T-scores and alpha angle in men with extensive and limited osteophyte development. **c** Analysis of age, BMI, T-scores and alpha angle in women with multiple and no osteophyte formation. **d** Quantification of age, BMI, T-scores and alpha angle in men with multiple and no osteophyte formation. **e** Quantitative joint space width analysis in relation to age, BMI, T-scores and alpha angle in women with narrow and wide joint space widths. **f** Joint space width analysis with regards to age, BMI, T-scores and alpha angle in men with narrow and wide joint space widths. Student’s *t*-test was used for normal distributed data and Mann-Whitney *U* test was used for nonparametric data. Exact *p*-values are reported for statistically significant comparisons unless *p*<0.001.
